# A Rare Case of Bone Marrow Sarcoidosis

**DOI:** 10.7759/cureus.63991

**Published:** 2024-07-06

**Authors:** Kejal Shah, Joud Enabi, Hema Kondakindi, Srikanth Mukkera

**Affiliations:** 1 Internal Medicine, Texas Tech University Health Sciences Center, Odessa, USA; 2 Rheumatology, Texas Tech University Health Sciences Center, Odessa, USA

**Keywords:** bone marrow, sarcoidosis, autoimmune disease, bone marrow sarcoidosis, rheumatology

## Abstract

Sarcoidosis is a systemic disease characterized by non-caseating epithelioid granulomas and can affect multiple organ systems, most commonly the lungs. Bone marrow involvement in sarcoidosis is rare and not well-documented. This case report details a 50-year-old female presenting with chronic lower back pain and significant hypercalcemia. Imaging revealed non-obstructive renal stones, liver nodularity, splenomegaly, and lung nodules. Initial treatments included corticosteroids for suspected sarcoidosis. Diagnostic workup, including bronchoalveolar lavage and various malignancy markers, was negative. A bone marrow biopsy showed non-caseating granulomas, confirming bone marrow sarcoidosis. This case underscores the importance of considering bone marrow involvement in patients with extrapulmonary sarcoidosis symptoms and highlights the need for a high index of suspicion among healthcare providers. Corticosteroids remain the primary treatment, with ongoing research into alternative therapies.

## Introduction

Sarcoidosis is a systemic condition characterized by non-caseating epithelioid granulomas [1]. First described by Norwegian dermatologist Caesar Boeck in 1899, the disease presents a wide array of clinical symptoms involving multiple medical specialties [2]. It is thought to arise from immune reactions triggered by various environmental factors [3,4]. In the United States, the annual incidence is about 10 cases per 100,000 individuals [5]. The condition most commonly affects individuals aged 20 to 39 years and occurs across all races and ethnicities [6]. In over 90% of cases, sarcoidosis affects the lungs [7]. Although extrapulmonary manifestations can be subtle, they contribute significantly to morbidity [8,9]. Bone marrow sarcoidosis is rarely documented among these manifestations. We present a case of sarcoidosis confirmed by lung and bone marrow biopsy.

## Case presentation

**Figure 1:**
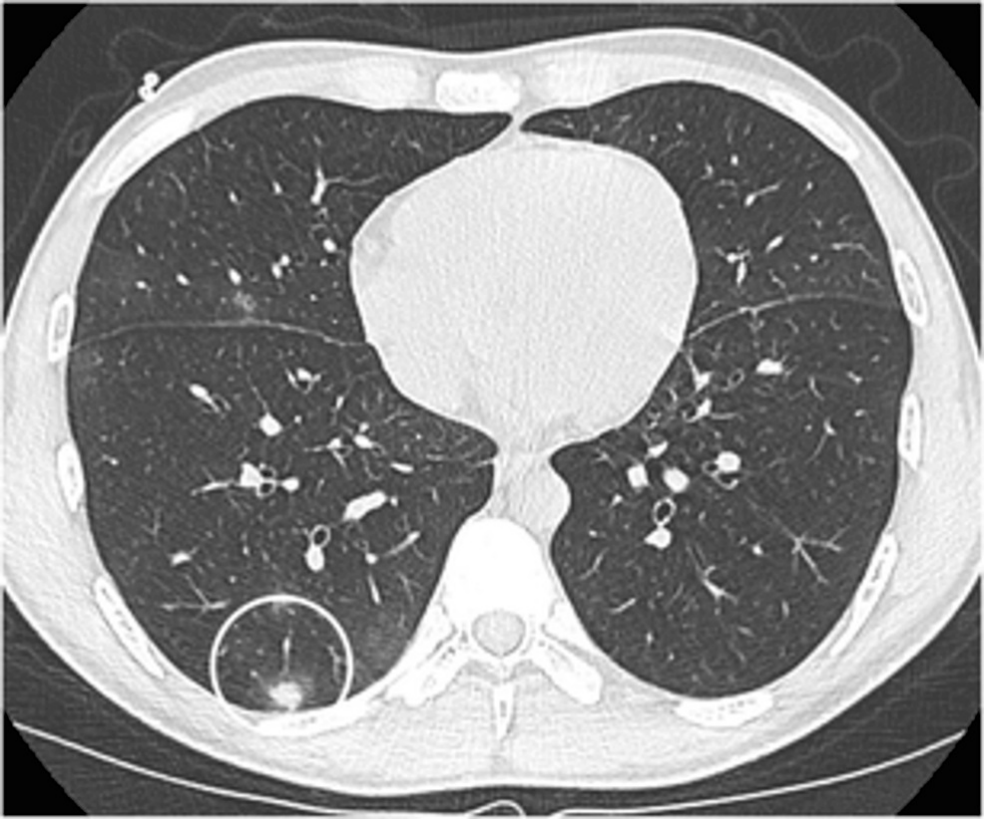
A CT chest scan showing scattered parenchymal, subpleural, and peri-fissural nodules.

A 50-year-old female presented with chronic lower back pain to her primary care physician. Routine blood work showed significant hypercalcemia (15.1 mg/dL), prompting a visit to the emergency room. She had no other complaints. Her medical history included hypertension, hyperlipidemia, diabetes mellitus, chronic kidney disease secondary to glomerulosclerosis and hypertensive nephrosclerosis (baseline creatinine 1.6 mg/dL), major depressive disorder, and generalized anxiety disorder. Her medications were tirzepatide 15 mg/0.5 mL weekly, allopurinol 100 mg daily, atorvastatin 10 mg daily, buspirone 5 mg daily, and escitalopram 20 mg daily, and vitamin D3 50,000 IU weekly. A CT scan of the abdomen and pelvis without contrast showed bilateral non-obstructive renal stones, mild liver surface nodularity, and mild splenomegaly (Figure 1).

The patient was treated for severe symptomatic hypercalcemia with normal saline at 200 cc/hour, calcitonin 400 units subcutaneously every 12 hours for four doses, and zoledronic acid. Further tests included normal serum protein electrophoresis/immunofixation results. She was started on corticosteroids for possible sarcoidosis.

Bronchoalveolar lavage was negative for malignant cells, showing respiratory epithelial cells and mixed inflammation. *Pneumocystis pneumonia* stain and acid-fast bacilli were negative. Angiotensin-converting enzyme was normal at 10 U/L. Mycology/Fungal panel was negative for beta-D-glucan, *Coccidioides* IgG and IgM, *Histoplasma*, and TB QuantiFERON. Malignancy markers were unremarkable with carcinoembryonic antigen of 1.46 ng/dL, cancer antigen 125 of 20.6 U/L, cancer antigen 19-9 of 21.7 U/L, and alpha-fetoprotein of <1.82 ng/mL. A nuclear medicine liver scan indicated decreased uptake of sulfur colloid by the liver’s Kupffer cells and increased uptake by the spleen, suggesting possible functional damage to the liver’s reticuloendothelial system. A liver biopsy showed benign hepatic parenchyma with bridging fibrosis, focal regenerative nodule formation, and no granulomas. There was mild portal chronic inflammation and minimal (less than 5%) macrovesicular steatosis. Bone marrow biopsy revealed significant replacement of marrow space by multifocal single and clustered epithelioid non-caseating granulomas with numerous multinucleated giant cells, suggestive of bone marrow sarcoidosis. Extrapulmonary manifestations of sarcoidosis are uncommon.

## Discussion

The precise cause of sarcoidosis remains unclear [10]. It can mimic other conditions and is primarily diagnosed through histopathology. The annual incidence of sarcoidosis varies by location, with the lowest rates in Japanese men and the highest in African American women [9,10]. Environmental exposures, specific human leukocyte antigen (HLA) alleles, and genetic factors play a role [10]. Sarcoidosis has been linked to exposure to irritants, inorganic particles, insecticides, and moldy environments [11]. Occupational studies have associated it with professions such as firefighting, metalworking, and Navy service [12]. Several gene products are implicated, including HLA-B8 antigens, HLA-DRB1 and DQB1 alleles, HLA-DQ and HLA-DR, HLA-DQB1*0201, and HLA-DRB1*0301 [12].

Sarcoidosis presents with intrathoracic and extrathoracic symptoms, varying based on age, gender, and ethnicity [13]. Bone marrow involvement is poorly characterized [13]. In a study of 640 sarcoidosis patients, predominantly Caucasians, none had bone marrow involvement at diagnosis, and only 0.3% developed it during follow-up [14]. The ACCESS study of 736 patients, primarily African Americans, found that 3.9% had bone marrow involvement [15]. Different ethnic groups show varying bone marrow sarcoidosis rates, higher in Mexico (23.4%) and none reported in China, Japan, and India [16]. Diagnosing sarcoidosis relies on a thorough medical history and physical examination. A complete blood count is recommended to assess bone marrow involvement [16]. Anemia is the most common hematologic abnormality, possibly due to iron deficiency, hemolysis, or anemia of chronic disease [2]. Anemia caused by bone marrow infiltration can be as high as 27% [17]. Traditional anemia assessments, such as serum ferritin, may not be reliable markers in sarcoidosis patients. Leukopenia can also be an initial sign of sarcoidosis resulting from bone marrow infiltration, hypersplenism, or lymphocyte redistribution [17]. Although rare, leukopenia can indicate severe disease [18]. Higher occurrences of anemia, leukopenia, and extrapulmonary involvement are seen in bone marrow sarcoidosis cases [18]. Diagnosing cytopenias in sarcoidosis involves a stepwise approach. Without hematologic abnormalities, diagnosing bone marrow sarcoidosis is challenging [18]. In our patient’s case, anemia and leukopenia were due to a combination of bone marrow involvement and hypersplenism. The estimated occurrence of granulomas in bone marrow biopsies is low, ranging from 0.3% to 2.2% in various studies. Sarcoidosis accounts for a significant proportion of these cases, with some reports indicating it could be as high as 21%. Clinical reasons for bone marrow biopsy in sarcoidosis patients are not well established. Our patient had cutaneous sarcoidosis, weight loss, generalized lymph node enlargement, and low red and white blood cell counts, prompting bone marrow analysis. Sarcoidosis can affect multiple organs, with 26% of patients experiencing splenomegaly, increasing with other extrapulmonary lesions. Neurological complications occur in 3-10% of sarcoidosis cases, with cranial neuropathy and meningeal disease being the most common. In bone marrow sarcoidosis, neurological symptoms are rare and typically linked to spinal cord compression. Lesions may be too small for imaging detection, explaining our patient’s tingling or numbness sensations. No randomized controlled trials have compared treatment approaches for bone marrow sarcoidosis. Prednisone is the primary treatment. Adalimumab may be considered when corticosteroids are unsuitable. Methotrexate, studied as a corticosteroid-sparing treatment in sarcoidosis, is used cautiously in bone marrow sarcoidosis due to its potential cytotoxic effects.

## Conclusions

Bone marrow sarcoidosis is a rare form of extrapulmonary sarcoidosis, more frequently seen in women and African Americans. Unexplained cytopenias, while not specific, might be the only noticeable symptom, hence healthcare providers should maintain a high level of suspicion. Before resorting to histopathological analysis for diagnosis, it is important to consider more common causes such as vitamin deficiencies, anemia related to chronic diseases, hemolysis, and hypersplenism. The primary treatment involves corticosteroids, with ongoing trials for promising alternative medications.
